# Ultrafast photonics applications of emerging 2D-Xenes beyond graphene

**DOI:** 10.1515/nanoph-2022-0045

**Published:** 2022-03-25

**Authors:** Huanian Zhang, Shuo Sun, Xinxin Shang, Bo Guo, Xiaohui Li, Xiaohan Chen, Shouzhen Jiang, Han Zhang, Hans Ågren, Wenfei Zhang, Guomei Wang, Cheng Lu, Shenggui Fu

**Affiliations:** School of Physics and Optoelectronic Engineering, Shandong University of Technology, Zibo 255049, China; Shandong Ruixing Single Mode Laser Technology Co. Ltd, Zibo 255049, China; School of Physics and Electronics, Shandong Normal University, Jinan 250014, China; Key Laboratory of In-fiber Integrated Optics, Ministry of Education, Harbin Engineering University, Harbin 150001, China; School of Physics & Information Technology, Shaanxi Normal University, Xian 710119, China; School of Information Science and Engineering, Shandong Provincial Key Laboratory of Laser Technology and Application, Shandong University, Qingdao 266237, China; College of Physics and Optoelectronic Engineering, Shenzhen Key Laboratory of Micro-Nano Photonic Information Technology, Guangdong Laboratory of Artificial Intelligence and Digital Economy (SZ), Shenzhen University, Shenzhen 518060, China; School of Chemistry, Biotechnology and Health Department of Theoretical Chemistry and Biology, KTH Royal Institute of Technology, Stockholm, Sweden

**Keywords:** saturable absorber, single element 2D materials, ultrafast applications, Xenes

## Abstract

Driven by new two-dimensional materials, great changes and progress have taken place in the field of ultrafast photonics in recent years. Among them, the emerging single element two-dimensional materials (Xenes) have also received much attention due to their special physical and photoelectric properties including tunable broadband nonlinear saturable absorption, ultrafast carrier recovery rate, and ultrashort recovery time. In this review, the preparation methods of Xenes and various integration strategies are detailedly introduced at first. Then, we summarize the outcomes achieved by Xenes-based (beyond graphene) fiber lasers and make classifications based on the characteristics of output pulses according to the materials characterization and nonlinear optical absorption properties. Finally, an outlook of the future opportunities and challenges of ultrafast photonics devices based on Xenes and other 2D materials are highlighted, and we hope this review will promote their extensive applications in ultrafast photonics technology.

## 1 Introduction

Since the first demonstration of laser in 1960, ultrashort pulse lasers attracted much attention and promoted the progress of the fields like modem iatrology, biology, chemistry, material processing, and military [1], [2], [3], [4], [5]. Especially, inspired by the invention of Ti-sapphire mode-locked lasers, ultrafast lasers provide a steady and reliable optical source for numerous basic and foreword scientific research [6]. In recent decades, benefiting from the advantages of excellent beam quality, high conversion efficiency, compact structure, free alignment, excellent heat dissipation and environmental robustness, ultrafast fiber lasers have been fundamental tools in the fields of advanced materials processing, medical diagnosis and treatment, optical communication, laser radar, nonlinear microscopy and so on [7], [8], [9], [10], [11], [12]. Mode-locked technique is the basic method to generate ultrashort picosecond (ps) or femtosecond (fs) pulses. Compared with actively mode-locked fiber lasers based on electro-optic modulator (EOM) or acousto-optic modulator (AOM), passively mode-locked fiber lasers get more attention due to the advantages of environmental stability, compact structure, easy integration, low cost, and high efficiency [13], [14], [15]. Saturable absorber (SA) devices are the kernel of passively mode-locked technique, which can be divided into artificial SAs and real SAs [16], [17], [18], [19], [20]. Artificial SAs can realize the nonlinear absorption related to the intensity of incident light based on birefringent properties, dependent rotation of an elliptical polarized light or nonlinear refractive index, such as Kerr lens (more often used in free-beam systems), nonlinear polarization rotation (NPR), and nonlinear amplifying loop mirror (NALM) [21], [22], [23], [24], [25]. However, enslaved to poor environmental stability, low output power and difficult to self-start, NPR, and NALM without using polarization maintaining fiber have not gotten wide applications. And those NPR and NALM-based lasers with special cavity structure (such as Figures 8 and 9 cavity) and polarization maintaining fiber will inevitably increase the cost, volume and structural complexity. Semiconductor saturable absorber mirrors (SESAMs) are the typical representatives of real SAs and have been used in commercial mode-locked fiber lasers. Nonetheless, the complex fabrication and encapsulation process, cumbersome alignment and high cost greatly cut down the advantages of all-fiber format.

Nanotechnology and materials science are innovating constantly along with the development of laser technology; advances in nanoscale materials manufacturing technology exploit novel possibilities in the fabrication of new materials. In recent decade, low dimensional materials, as another type of real SAs with intense nonlinear saturable absorption effect, ultrafast carrier recovery time, and simpleness of preparation and integration to fiber systems, have opened up a new way for the design of photonic devices, such as 0-dimensional (0D) quantum dots (QDs), and 1D carbon nanotubes (CNT) a lot of researches have been reported [26–33]. In addition to these, although the researches of 2D layered materials have lasted for over 150 years, the enormous research interest really picked up was in 2004 that single-atom-thick graphene was first exfoliated from graphite by Novoselov et al. [34], [35], [36]. Since then, due to the abundant fascinating electrical, optical, and chemical characteristics like atomic layer thickness, high-carrier mobility, high optical absorption coefficient and strong light-material interaction, plenty of researches about photonic applications based on graphene and other 2D materials have been reported [37], [38], [39], [40], [41], [42], [43], [44], [45], [46]. Among these 2D materials, graphene is the pioneer in applications of ultrafast photonics devices. However, the zero-bandgap structure limits its applications in situations required strong light–matter interaction [47]. Black phosphorus (BP) considered to be an ideal SA with high charge carrier mobility, tunable bandgap value of 0.3–2 eV and unique in-plane anisotropic structure [48]. The bandgaps of transition metal dichalcogenides (TMDs) can cover the energy range from 0.86–2.5 eV, corresponding to the spectral range from visible to near-infrared [49], [50], [51]. Such as, the bandgaps of three well-known TMDs, which include MoS_2_, WS_2_, and SnS_2_, are 1.29, 1.35 and 2.24 eV for bulk structures, 1.9, 1.3 and 1.57 eV for monolayer [52], [53], [54]. However, the electronic and optical characteristics of TMDs highly depend on the number of layers, which limits the practical applications for photonic devices. Topological insulators (TIs) have a bandgap of 0.2–0.3 eV, can realize the output wavelength less than 4.2 μm [55, 56]. And the nonlinearity of TIs is better than graphene. But the slower relaxation time, complex preparation process and low damage threshold limit their development. Perovskites are novel materials for nonlinear optical research, but the existence of Pb element is harmful to our health. As a new member of 2D materials family, the nonlinear refractive index of transition-metal carbides and/or nitrides (MXenes) is about 10^−4^ cm^2^/W, which is larger than that of graphene of 10^−5^ cm^2^/W. But the preparation of MXenes is mainly by selective acid etching of the raw MAX phase, which is complex and high cost [57]. In addition to the 2D materials mentioned above, there are some emerging 2D materials attracting considerable attention due to their differentiation advantages, for example, ferromagnetic insulator (C_r2_Si_2_Te_6_, Cr_2_Ge_2_Te_6_), yttrium oxide (Y_2_O_3_) [58], [59], [60], and even some saturable absorbers with special structures based on fiber [61].

**Figure 1:**
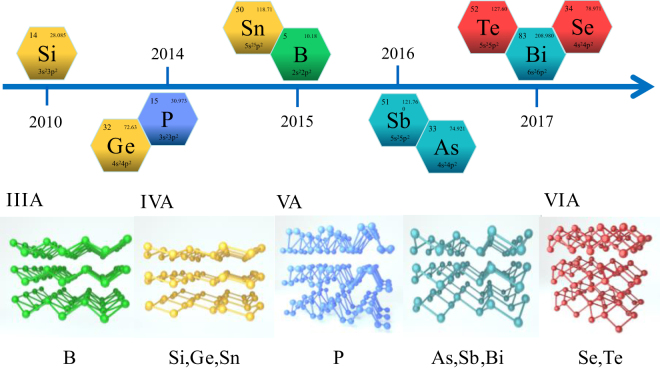
The most stable atomic structures of various main group mono-elemental 2D materials.

In recent years, composed with single element, an emerging subclass of 2D materials called 2D mono-elemental graphene-like materials (Xenes) have attracted intense interest [62], [63], [64], [65], [66], [67], [68], [69]. Xenes refer to the mono-elemental 2D materials with atomic thickness, X stands for the possible group elements from IIIA to VIA, and “ene” is a Latin word that denotes nanosheets [70]. Profiting from their outstanding characteristics of tunable bandgap, ultrahigh surface-volume ratio, folded structure, and high carrier mobility, Xenes have raised great interest in various fields. Particularly, Xenes from group IV to VI like silicene, germanene, stanene, phosphorene, arsenene, antimonene, bismuthene, selenene, and tellurene have been proven to have some differences from the other 2D materials [71], [72], [73], [74]. Figure 1 illustrates the most stable atomic structures and of various main group Xenes materials and a timeline of experimental realization of several recent elemental 2D materials after the isolation of graphene. By changing the number of layers, the bandgap of Xenes can be designed flexibly which is beneficial to optical sensing [75], [76], [77]. In the field of biomedicine, the properties of excellent biodegradability and ultrahigh surface-volume ratio make Xenes a good choice [78], [79], [80], [81]. Benefit from the in-plane anisotropy resulted in the fold structure, Xenes open a new door for the neural networks [82, 83]. Besides, the high mechanical strength is meaningful for the improvement of battery safety, the fold structure and ultrahigh surface-volume ratio are of great significance to improve the battery capacity [84], [85], [86], [87], [88], [89]. Xenes have shown great potential and have huge application prospect in addressing challenges in the fields of energy, environment, and healthcare.

Compared to other 2D materials, Xenes exhibit outstanding nonlinear absorption properties, excellent photoelectric response, high nonlinear coefficient, and strong anisotropy [90], [91], [92], [93]. Tunable layer-dependent bandgaps determine the possibility of 2D Xenes can realize the broadband absorption from ultraviolet to near-infrared and even mid-infrared, and fill the gap between zero-bandgap graphene and other large-bandgap 2D materials [57]. Similar to graphene, 2D Xenes have high Fermi velocity, linear dispersion electronic characteristics, strong spin-obit coupling, and open bandgap at the Dirac point [79]. Besides, by adjusting the substrate interaction and interlayer twisting, the properties of photoelectricity can be turned flexibly [94]. Based on the above characteristics, Xenes may have the potential to realize some breakthroughs in the applications of optoelectronics and photonics that other 2D materials can not get.

Over the past few years, Xenes have been successfully demonstrated in designing ultrafast photonics devices. There have been many reviews of graphene as saturable absorber, such as, ultrafast lasers mode-locked by nanotubes and graphene [95], graphene-based saturable absorber for pulsed fiber laser generation [96], graphene saturable absorbers applications in fiber lasers [97], so our review does not summarize the works on graphene saturable absorbers. In this paper, we review the development of 2D Xenes based ultrafast applications. Catalog the preparation methods of Xenes and various integration strategies are detailedly introduced at first. Then, according to the materials characterization and nonlinear optical absorption properties we summarize the outcomes achieved by Xenes-based lasers and make classifications based on the characteristics of output pulses. At last, an outlook of the future opportunities and challenges of ultrafast photonic devices based on Xenes and other 2D materials are highlighted.

## 2 Fabrication and characterization of 2D materials

### 2.1 Fabrication techniques

**Figure 2:**
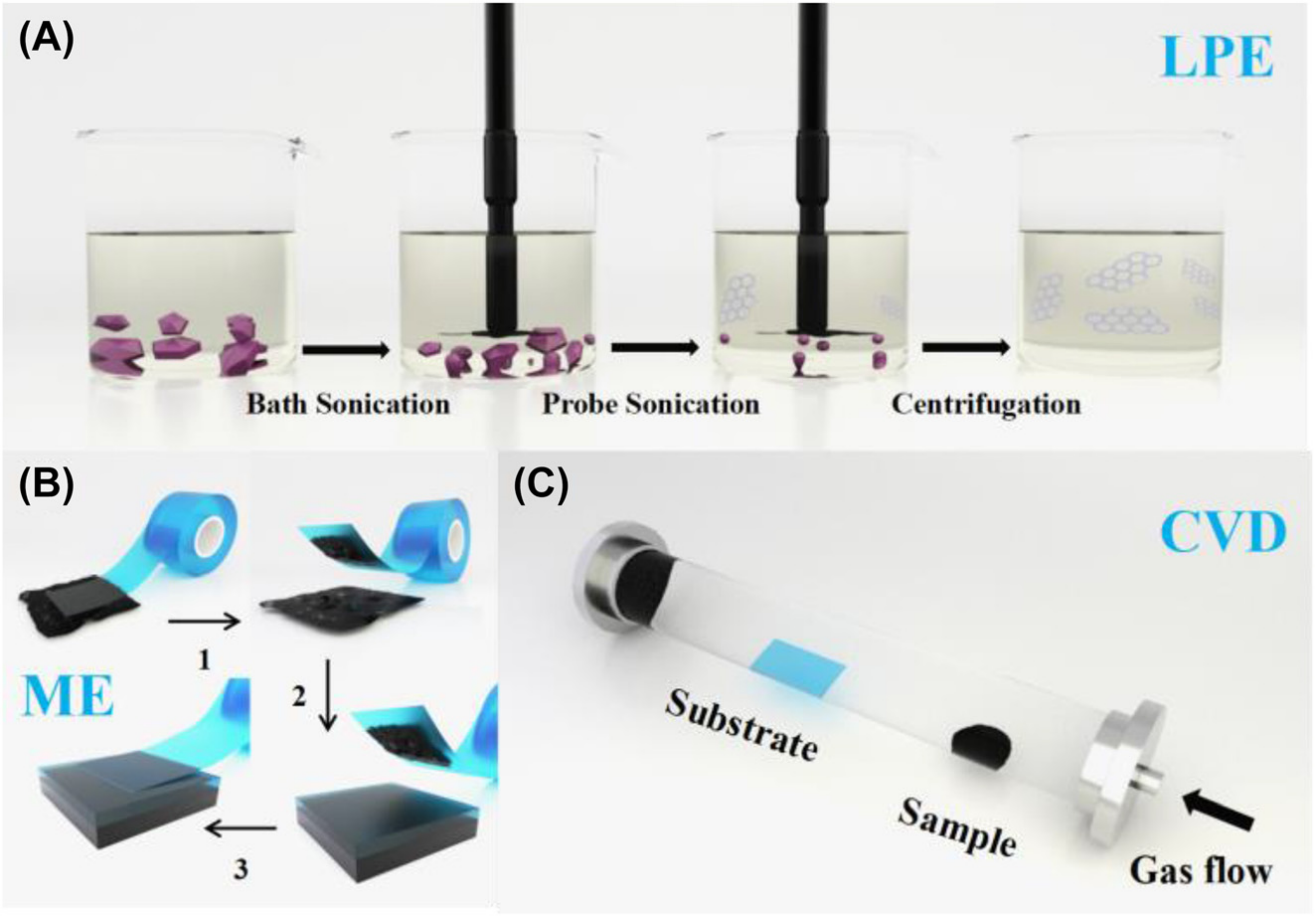
Schematic of fabrication techniques of 2D materials. (A) LPE method. (B) Mechanical exfoliation (ME) method. (C) CVD method.

Few-layer nanomaterials fabrication methods broadly include two directions: bottom-up of molecular precursors and top-down of bulk layered materials. Bottom–up growth methods refer to the film of molecules arranged in a single or several layers, such as chemical vapor deposition (CVD) and pulsed laser deposition (PLD). Top–down exfoliation methods (including mechanical cleavage and solution processing techniques) depend on the weak van der Waals interaction of layered nanomaterials, which makes it easy to strip nanosheets from large bulk layered materials. Figure 2 shows three mainly fabrication techniques of 2D materials.

CVD is a method of 2D nanomaterials growth by injecting precursor gas into the chamber with the heated substrate and making a chemical reaction on the heated substrate surface, a thin film is deposited from the steam in the end. The properties of the prepared material are affected by substrate material, substrate temperature, pressure, and gas flow. The characteristics of CVD technology are products of uniform thickness and low porosity even on substrates of complex shapes, and a wide variety of deposited materials. In addition, the use of distillation technology contributes to the purity of nanomaterials deposition very high. Micromechanical exfoliation method by using mechanical force to two-dimensional nanomaterial to stripped layered 2D material from bulk layered materials. For example, graphene was separated from graphite by the adhesive force of tape. Electrochemical exfoliation is a way of using electrical current as a driving force to carry foreign molecules or ions into a bulk layered material in the electrochemical cell, which will increase the interlayer distance and make 2D materials easier to strip. Liquid-phase exfoliation (LPE) method has been a general technology for preparing layered nanosheets from 2D materials, including graphene, transition metal dichalcogenides (TMDs), ferromagnetic insulators (FIs), transition metal oxides (TMOs), 2D mono-elemental graphene-like (Xenes). LPE refers to suspending layered crystals in a “solvent” and impacting the crystals with ultrasonic energy to break them into 2D layered materials. The general process of preparing 2D material nanosheets by LPE method involves following several steps: first, dispersion of the 2D material power in a liquid medium, then, the suspensions are employed sonication to assist the dispersal of nanosheets, last, centrifugation to remove the precipitation to get stable suspensions. The LPE method is an effective method for the preparation of large scale, highly concentrated atomically thin 2D nanosheets with the advantages of high quality and easy preparation. Saturable absorbers fabricated by ME, LPE, CVD, pulsed laser deposition (PLD), and magnetron sputtering deposition (MSD) are more convenient, easier and lower cost. ME and LPE belong to the top-down methods, they are the simplest methods to get nanosheets. Because of that polyvinyl alcohol (PVA) and polymethyl-methacrylate (PMMA) have low melting point, SAs fabricated by LPE with PVA or PMMA are usually stable under different environment and high thermal. However, the number of layers obtained by these two methods is random and uncontrollable. In contrast, the layer number of nanosheets prepared by the bottom-up methods (CVD, PLD, MSD) is controllable, especially the CVD method. Besides, the MSD method has been widely used in the fabrication of SA based on tapered fiber and D-shaped fiber. Finally, we note that there are other few layer 2D materials fabrication techniques were adopted to produce SA devices, such as sonochemical exfoliation, physical vapor deposition, and gas phase growth.

### 2.2 Characterization of Xenes materials

In the experiment, characterization methods, scanning electron microscopy (SEM), energy-dispersive X-ray spectroscopy (EDS), Raman, X-ray diffraction (XRD), transmission electron microscopy (TEM), high-resolution transmission electron microscopy (HR-TEM), and atomic force microscopy (AFM), were usually employed to test the surface and physical properties of 2D materials to better understand its nonlinear optical absorption properties.

**Figure 3:**
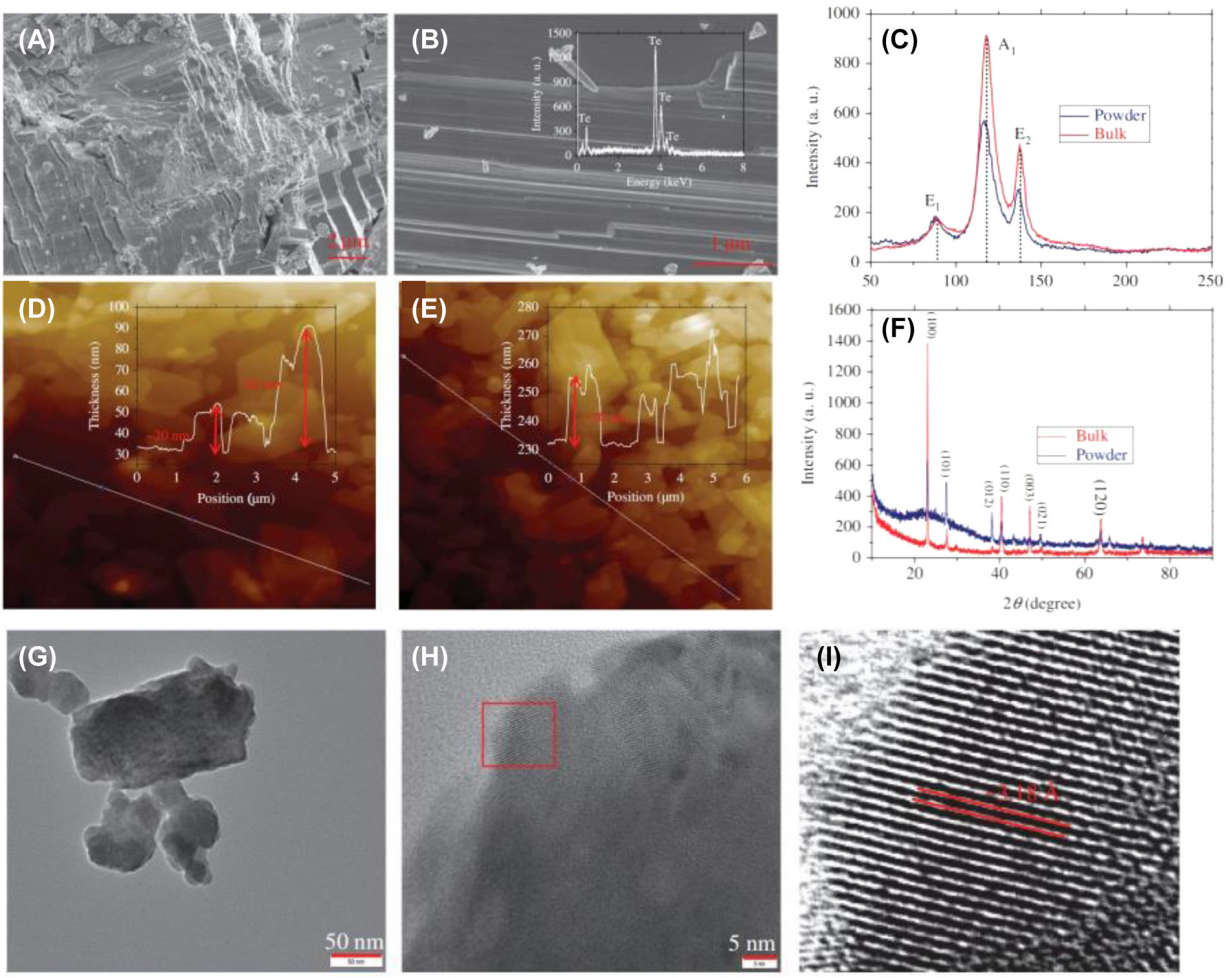
Characterization methods for tellurene. (A and B) SEM image, inset: EDS spectra. (C) Raman spectra. (D and E) AFM image. (F) XRD spectra. (G) TEM image. (H and I) HR-TEM image. Reproduced with permission [98].

The SEM is adopted for testing the surface morphology of the 2D materials. The layered structure can be seen if the materials are successfully fabricated. The thickness of nanomaterials can be calculated by AFM, we can estimate the number of layers further. The EDS spectra can provide the atomic ratio of materials. Raman and XRD spectra are used to detect the element composition and crystal structure. Additionally, to test the structure characteristics, TEM and HR-TEM are employed. For example, Figure 3 illustrates the characterization methods for tellurene. Obvious layered structure can be seen according to Figure 3A and B, Raman and XRD spectra also corresponds very well. Lattice fringes of tellurene are existed by HR-TEM image. All results indicate that pure tellurene nanosheets with a well layered-structure and high-crystallinity are prepared successfully.

## 3 Nonlinear optical properties of 2D materials

**Figure 4:**
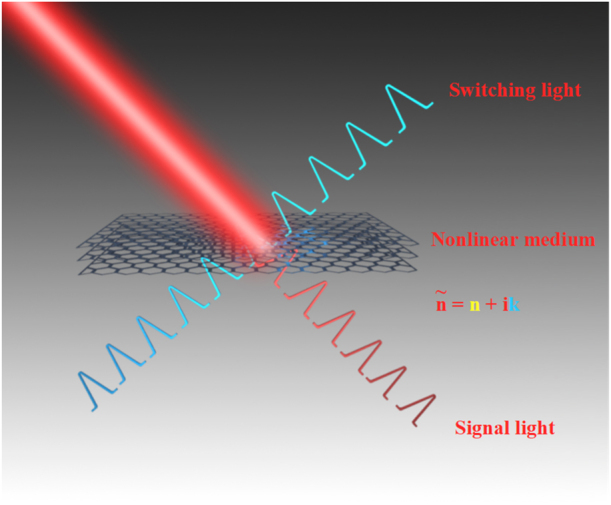
Schematic diagram of all-optical modulation. Switching light beam control and modulate the signal light. The light–light interaction is achieved by nonlinear medium, and the kernel of the all-optical modulation is to control or modulate the real or imaginary parts of the refractive index of the nonlinear medium.

In generally, the optical response of materials can be expressed by the refractive index as $\tilde{n}=n+i\beta$ , where *n* is the refractive index and β is the absorption characteristic. The refractive index can be changed by the light of amplitude modulation, phase modulation, polarization modulation, and so on. As shown in Figure 4, the signal light beam is controlled and modulated by a switching light beam, and the interaction between light and light is realized by the optical nonlinear effect of the nonlinear medium. When the laser pulse is excited, the carrier density and distribution of 2D materials may suddenly oscillate violently, which will change the refractive index and absorption characteristics. Optical nonlinearity effect plays an important role in ultrafast photonics and optoelectronic applications. The optical Kerr effect and saturable absorption are used to describe the nonlinear effects of 2D materials.

### 3.1 Optical Kerr effect

The optical Kerr effect is a third-order nonlinear phenomenon, which is manifested in the change of instantaneous refractive index of nonlinear medium caused by the incident light, which increases with the increase of incident light intensity. It can be expressed as:

(1) $$n=n_{0}+n_{2}I$$

Where $n_{0}$ is the linear refractive index, $n_{2}$ is the nonlinear refractive index, and *I* is the incident light intensity. The optical Kerr effect can lead to many nonlinear phenomena, such as self-phase modulation, cross-phase modulation, self-focusing, four-wave mixing, and modulation instability. 2D materials have special Kerr nonlinearity and have great potential in optical modulation.

Typical Z-scan measurement by using ultrashort pulses is an important means to detect the nonlinear absorption characteristics of 2D mono-elemental graphene-like (Xenes) materials. The thin medium with negative nonlinear coefficient can be regarded as a thin lens with variable focal length when a Gaussian beam is incident, and the focus of the lens is taken as the origin of the *Z*-axis. When the nonlinear medium moves from – Z to the origin, the initial light intensity is low, and the light refraction caused by nonlinearity can be ignored. Therefore, the transmittance *T* measured at aperture *A* remains relatively unchanged, which the linear transmittance is unchanged of the system. When the sample is scanned near the origin, the light refraction effect caused by nonlinearity significantly strengthened due to the increase of light intensity. At this time, the sample is equivalent to a negative lens to collimate the beam, narrowing the beam at aperture *A*, resulting in an increase in the measured transmittance. Therefore, at the side of Z < 0 close to Z = 0, the *T*–*z* curve shows a peak. When the nonlinear medium moves past the origin 0, the self defocusing effect of the sample will widen the beam at aperture A and reduce the transmittance. Similarly, the self defocusing effect is most obvious near *z* = 0, resulting in a valley in the *T*–*z* curve; At the origin, the far-field transmittance is the same as the linear value. From the above analysis, the Z-scan curve can be qualitatively obtained, which is in the shape of peak before valley.

**Figure 5:**
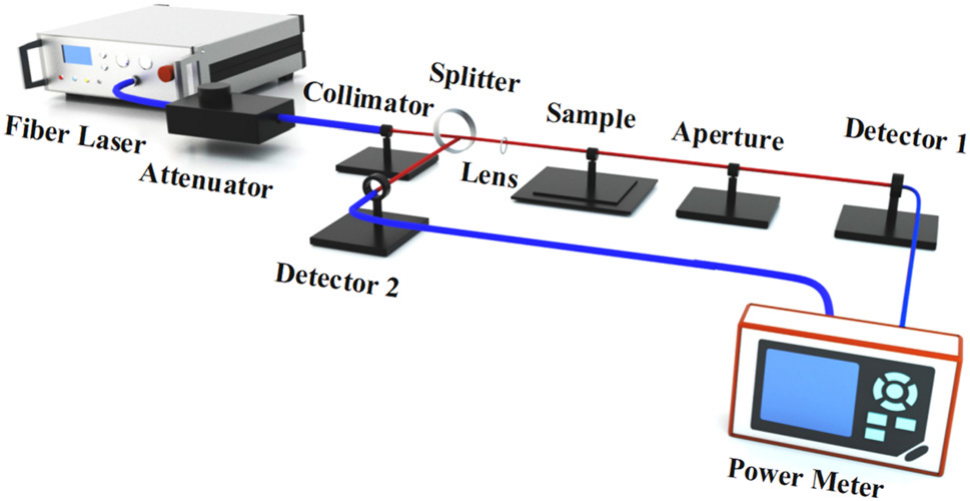
Schematic of Z-scan experimental setup.

For Z-scan measurement, as shown in Figure 5, at first, the two-dimensional materials were placed on the *z*-direction translation table, the light of the ultrashort pulse light source is divided into two parts. One path uses the low optical power measured by the slow detector as reference. Another path consists of high optical power part for materials characterization. Then, the light beam in the measurement part is focused on the 2D materials through the lens. The transmitted light passes through the two-dimensional materials and collected by the second detector. By comparing the readings of the two detectors, the strength dependent transmission characteristic of two-dimensional materials can be obtained. The *Z*-scan curve can be fitted with the following formula:

(2) $$T\left(z\right)=\left[1-\frac{\alpha_{0}LI_{s}}{I_{s}+I_{0}/\left(1+Z^{2}/Z_{0}^{2}\right)}\right]/\left(1-\alpha_{0}L\right)$$

according to the *Z*-scan theory [99]. In which, *Z* is the position of the sample relative to the focus, *Z*_0_ is the diffraction length of the beam, *α*_0_ *L* is the modulation depth, *T*(*z*) is the normalized transmittance at *Z*, *I*_0_ is the peak onaxis intensity at the focus, and Is the saturable intensity. However, the nonlinear absorption coefficient, which is significant to the description of nonlinear effects, is unknown only according to Eq. (2). So, another equation is needed to obtain the nonlinear absorption coefficient as following [100]:

(3) $$T\left(z\right)=1-\frac{\beta I_{0}L_{\mathrm{eff}}}{2\sqrt{2}\left(1+z^{2}/z_{0}^{2}\right)}$$

where *β* and *α*_0_ represent the nonlinear and linear absorption coefficient, respectively, and *L*_eff_ is the effective length which can be expressed by the following formula [101]:

(4) $$L_{\mathrm{eff}}=\left(1-e^{-\alpha_{0}L}\right)/\alpha_{0}$$

The nonlinear optical properties of 2D Xenes materials measured by the Z-scan technology are summarized in Table 1. In 2018, Zhang et al. used Z-scan to explore the nonlinear absorption characteristics of silicene for the first time [103]. Under 532 nm excitation, silicon has low saturation intensity and large two-photon absorption coefficient. Experimental results show that silicon has potential application prospects in ultrafast lasers and optical limiting devices. The measurement of nonlinear optical properties is of great significance for the optical applications of 2D materials.

**Table 1:** Summarization of nonlinear optical properties of 2D Xenes.

2D materials	Laser parameters	*T*(%)	*I*_*s*_ (GW/cm^2^)	Im*χ* ^(3)^ (esu)	Ref.
Antimonene	1064 nm, 10 Hz, 40 ps	18.4	1.3	–	[102]
Silicene	532 nm, 10 Hz, 30 ps	–	6.9	3.7 × 10^−5^	[103]
Bismuthene	2 μm, 5 kHz, 70 ns	15.4	183.9 kW/cm^2^	–	[104]
Tellurene	515 nm, 1 kHz, 350 fs	3.2	11.2	2.383 × 10^−22^	[105]
Tellurene	1550 nm	27	78.14	–	[106]
BP	800 nm, fs	36	3.3	–	[107]
BP	1.5 μm, 20 kHz, 190 fs	12.4	2.16 μJ/cm^2^	–	[108]

*T*: modulation depth; *I*_*s*_: saturation intensity; Im*χ* ^(3)^: third-order nonlinear susceptibility.

### 3.2 Saturable absorption properties

**Figure 6:**
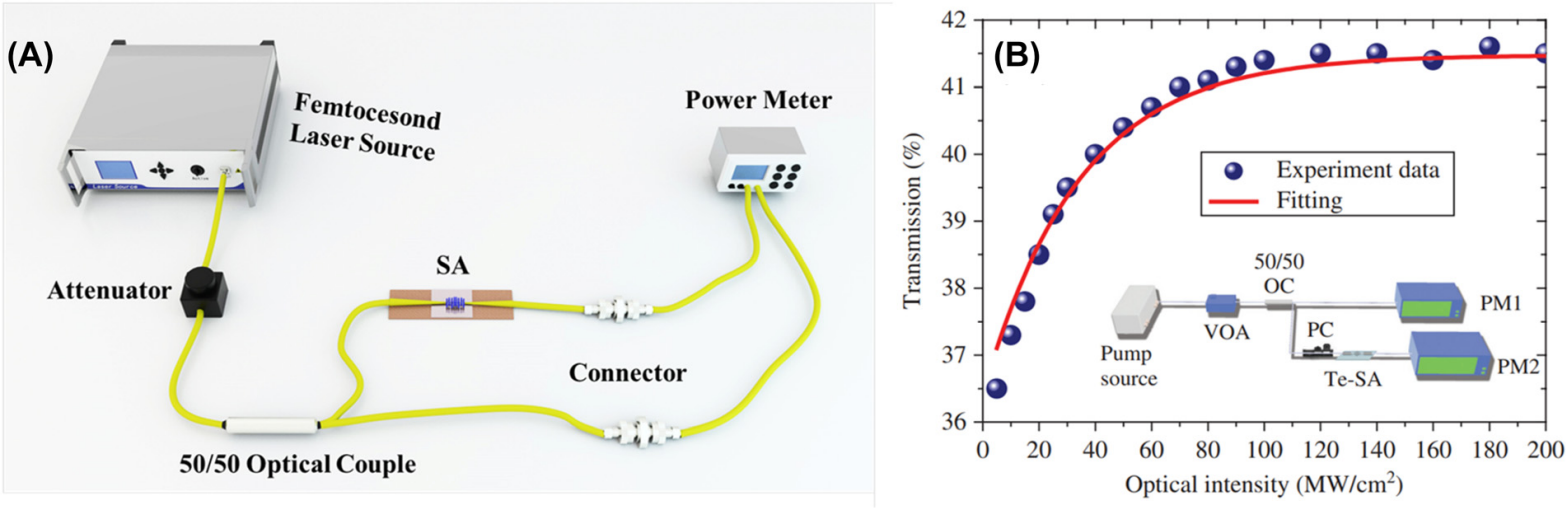
Nonlinear optical characterization: (A) Schematic of dual arm detection system. (B) Power-dependent nonlinear optical property of tellurenebased SA. (Inset) Corresponding experimental setup for testing nonlinear optical property. Reproduced with permission [98].

Dual arm detection system can measure the nonlinear absorption characteristics of the SA-based D-type fiber, tapered fiber, and SA in optical fiber devices. As shown in Figure 6A, the ultrashort pulse is divided into two paths through the output coupler with a coupling ratio of 50:50. One path reaches the power meter by connecting an optical fiber containing the SA. The other path is directly connected to the power meter. The obtained data are fitted by the following formula:

(5) $$T\left(I\right)=1-T_{\mathrm{ns}}-\Delta \cdot \text{exp}\left(-\frac{I}{I_{\mathrm{sat}}}\right)$$

Where, T(I) and Δ are the transmission velocity and modulation depth, respectively. I and $I_{\mathrm{sat}}$ correspond to incident light intensity and saturation energy, $T_{\mathrm{ns}}$ is nonsaturable absorption. As shown in Figure 6B, the saturation intensity, nonsaturable loss, and modulation depth of the tellurene SA are 34.3 mW/cm^2^, 58.6%, and 5.0%, respectively [98].

**Figure 7:**
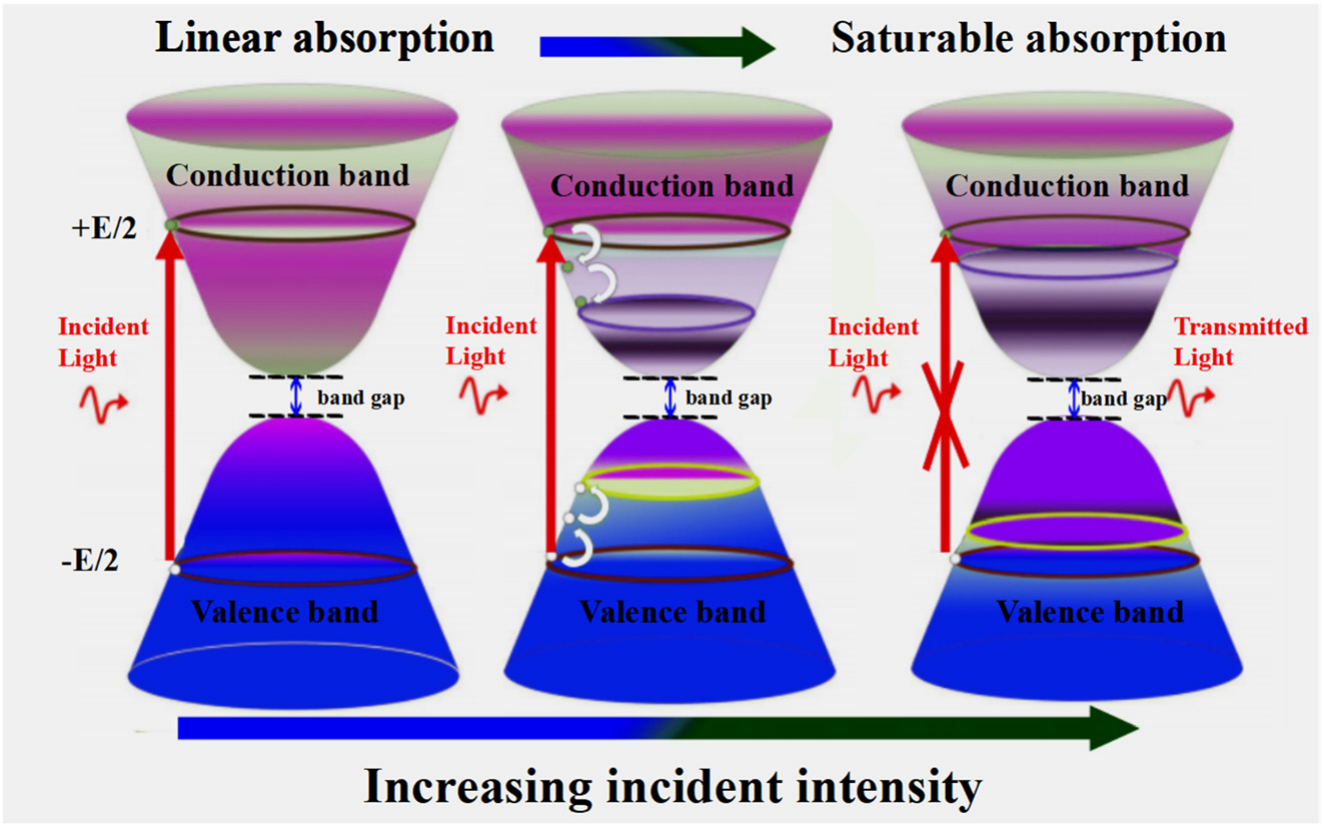
The process of linear absorption and saturable absorption of Xene due to Pauli blocking effect: (left) interband transition of the electron due to light excitation; (middle) hot carriers lend to thermal balance; (right) blocking of absorption for light.

The saturable absorption property is the core mechanism of 2D materials used as ultrafast photonics devices in mode-locked or Q-switched lasers. Similar to other 2D materials, the process of saturable absorption of mono-elemental graphene-like (Xenes) can also be interpreted by the Pauli blocking principle, which is shown in Figure 7. Under normal conditions, most electrons are in the valence band, this balance will not be broken until the light is incident on the SA. Linear absorption will occur firstly if the intensity of incident light is low. When the incident light has larger energy than the bandgap value of the SA, electrons in the valence band will be excited into the conduction band by absorbing the energy of photons (the energy of one photon is ℏω ). After that, these hot electrons generated by photons will cool down to constitute a hot Fermi–Dirac distribution in an extremely short time which is about ps or fs magnitude. At this moment, the newly formed electron–hole pairs will suppress some originally potential interband photon transitions and absorption. Then, electron–hole pairs recombine until a new equilibrium is formed under the intraband phonon scattering. However, the photocarriers will increase instantaneously and fill the energy states near the edge of the conduction and valence band when the intensity of incident light increases to a higher level. In this case, the absorption will be blocked under the constraint of Pauli blocking principle that no two electrons can fill the same state. And now, specific frequency photons could transmit the SA without loss. Thus, the 2D materials with appropriate bandgap value is of great value for the design of ultrafast photonics devices.

## 4 SA incorporation methods

**Figure 8:**
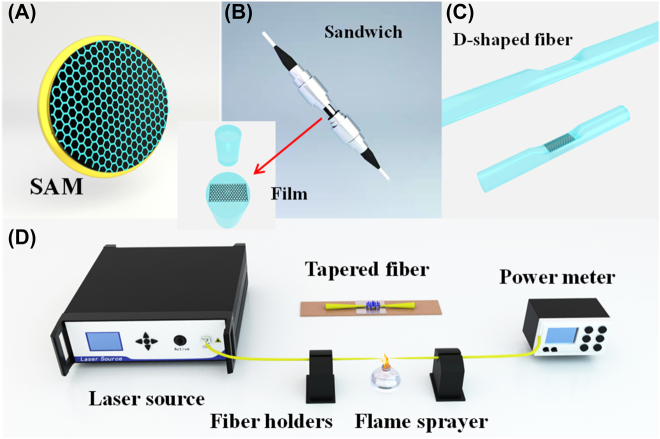
Integration schemes to form nanomaterial-based photonic devices.

The methods for preparing SA based on 2D materials mainly include SA mirror (SAM), deposited on the fiber endface, tapered fiber, D-shaped fiber, and polymer materials composite films. As show in Figure 8A, SAM is prepared by thin film of 2D material and transferring the film onto a mirror substrate. Such as semiconductor saturable absorber mirrors (SESAMs), single wall carbon nanotube SAM (SWCNT-SAM), graphene SAM. Semiconductor saturable absorber mirrors (SESAMs) are the typical representatives of real SAs and have been used in commercial mode-locked fiber lasers. Nonetheless, the complex fabrication and encapsulation process, cumbersome alignment and high cost greatly cut down the advantages of all-fiber format. 2D materials polymer materials composite thin film SA attached to the end face of the fiber connector shown in Figure 8B. Thin-film SA like 2D materials-PVA films are easy to transfer, but they can’t withstand high-power or long-time beam lighting. By depositing 2D-materials nanosheets on D-shaped or tapered fiber can improve this problem, and the intrinsic nonlinear effects can produce more abundant and interesting optical phenomena. It is more attractive that D-type optical fiber is used to prepare SA. D-type optical fiber has a larger fiber diameter and higher robustness due to it generally made by placing arc-shaped blocks on single-mode fiber and polishing it. D-type optical fiber with 2D materials deposited on its surface is shown in Figure 8C. D-shaped fiber SA is based on the evanescent field between the 2D materials and optical field, it has longer nonlinear optical interaction length and higher damage threshold than the 2D materials-film SA. Tapered fiber SA is also based on the evanescent field between the 2D materials and optical field. Figure 8D shows experimental setup of preparing the tapered fiber, tapered fiber is fabricated by standard single-mode fiber are stretched in a molten state heated by a flame. The diameter and length of the fiber are gradually reduced to a suitable range as the conical fiber is stretched. Both ends of the tapered fiber are coupled to a semiconductor laser and a power meter to measure the loss of the prepared tapered fiber. The inset of Figure 8D presents the SA by the tapered fiber deposited with 2D material. More abundant and interesting optical phenomena can be obtained due to the long nonlinear optical interaction length and higher damage threshold. But D-shaped or tapered fiber based SA will increase the difficulty of the preparation process and increase the cost. Another SA preparation method is that 2D material deposited on the fiber end face by laser radiant thermal deposition. The nonlinear response of the tapered fiber SA and D-type fiber SA is realized through the nonlinear interaction between the evanescent field on the fiber surface and the 2D material deposited in the tapered region. The nonlinear effect is depend on the interaction length and damage threshold between laser and layered materials deposited on the surface of tapered region, which is of great significance to the design of better ultrafast photonic devices. Compared with SESAMs, the other incorporation methods have their own different advantages and disadvantages. The application of SA material on special optical fibers has become a research hotspot.

## 5 Demonstration of ultrafast lasers based on Xenes beyond graphene

**Figure 9:**
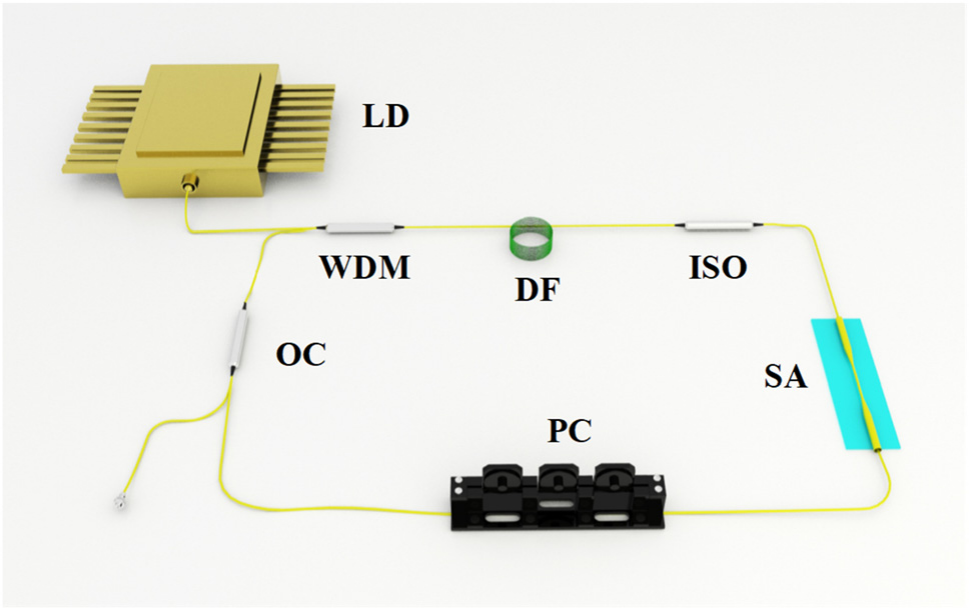
Typical ultrafast fiber laser system using 2D Xenes as SA.

Figure 9 shows the typical ultrafast ring cavity fiber laser system based 2D Xenes as SA. A piece of gain fiber (GF) is pumped by the pump source which is a fiber-pigtailed laser diode (LD), the GF mainly include Yb-doped, Er-doped, Ho-doped and Tm-doped fibers. The operating wavelength of the laser can be changed from near-infrared band to mid-infrared band by selecting appropriate pump source and matching different GF. Besides, the polarization state and transmission direction of the laser in cavity are controlled by a polarization controller (PC) and a polarization-independent isolator (PI-ISO), respectively. Through an optical coupler with a certain output coupling ratio, the information of the output pulse can measured by a digital oscilloscope combined with a high-speed photodetector, an optical spectrum analyzer, a radio frequency (RF) spectrum analyzer, an optical power meter, and auto-correlator instrument, respectively. Up to now, various lasers have been developed by using the 2D Xenes materials as SAs. Then, we will briefly review these lasers as follows.

### 5.1 Q-switched operation

**Figure 10:**
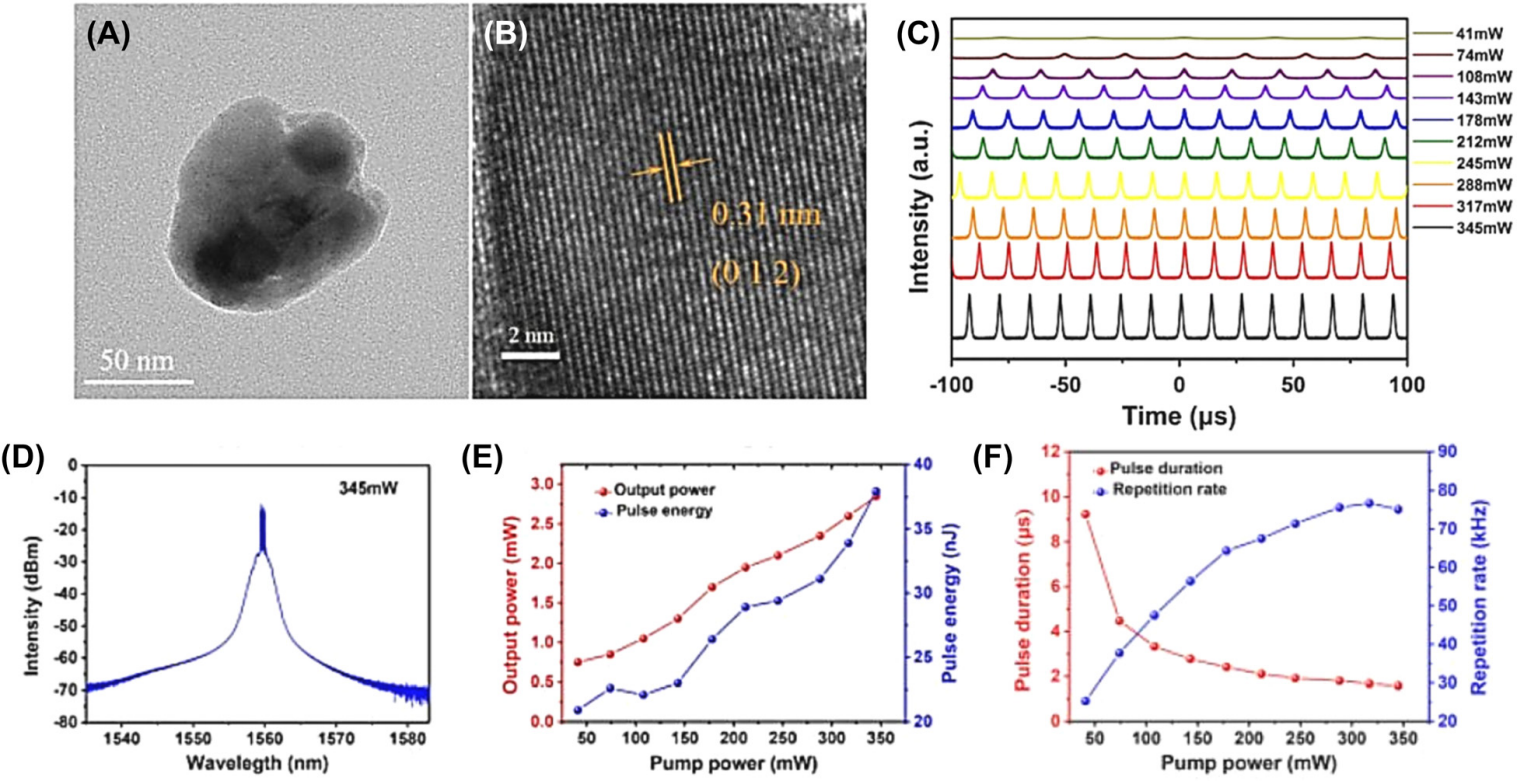
Material characterization: (A) TEM image. (B) HR-TEM image. Q-switching characteristics of an EDFL based on antimonene SA: (C) Pulse trains at different pump powers. (D) The optical spectrum. (E) output power and pulse energy versus the pump power. (F) Pulse duration and pulse repetition rate versus the pump power. Reproduced with permission [111].

Q-switched lasers have been widely applied in optical sensing, optical frequency metrology, laser marking, optical communications, and scientific research due to the advantages of robustness, high pulse energy, and excellent beam quality. The pulse energy of Q-switched laser can reach to μJ or mJ level with low repetition rate and narrow pulse width, which are generally kHz and ns level, respectively. In recent years, 2D Xenes nanomaterials beyond graphene based SAs have been widely used in passively Q-switched laser. As shown in Table 2, the performance of Q-switched lasers based on 2D Xenes materials is classified and summarized. Among them, the maximum pulse energy of 15.95 μJ and minimum pulse width of 48.33 ns were demonstrated [114, 125], respectively. As an example, Hu et al. achieved the stable Q-switched operation based on antimonene SA [111], the pulse repetition rate varies from 25.3 to 76.7 kHz when the pump power increased from 41 to 345 mW, the shortest pulse duration is 1.58 µs with pulse energy of 37.9 nJ, as shown in Figure 10. The Q-switched operating wavelength of 2D Xenes-based lasers cover from visible to mid-infrared. For example, Hai et al. demonstrated that topological semimetal antimonene possesses an ultrabroadband optical switch characteristic covering from 2 μm to beyond 10 μm, based on antimonene, passively Q-switched pulsed lasers at 2 and 3.5 μm wavelengths were realized [109]. In 2019, Zhang et al. designed a passively Q-switched Nd:LuAG laser with fluorinated antimonene, centered at 1064 nm with a pulse width of 326.7 ns and a repetition rate of 733.1 kHz demonstrating its potential application as SA [116]. In 1.3 μm, a passively Q-switched bulk laser with the maximum average output power of 125 mW and the shortest pulse width of 510 ns was demonstrated by Su et al. [119]. Recently, Han et al. Proved the visible nonlinear optical properties of tellurium and application as SA, they realized the passively Q-switched Pr: YLF laser over the spectrum of orange (605 nm), red (639 nm), deep red (721 nm) with tellurium film as SA [105]. In 2021, Hassan report on tellurium nanorods as SA induced four-wave-mixing to construct an all-fiber multi-wavelength passively Q-switched erbium-doped-fiber laser, simple and green chemical process were used to prepare 1D tellurium nanorods SA, experimental results show that the SA device simultaneously generates seven wavelengths within 1592.4–1599.6 nm waveband with mode spacing of 1.2 nm. There have been many reports on BP-based Q-switched lasers [126], [127], [128], [129], [130], [131], [132], [133], [134], [135], [136], [137], [138], [139], [140], [141], [142], [143], [144], [145], [146], [147], [148], [149], [150], [151], [152], [153], [154], [155], [156], [157], [158], [159], [160], [161], [162], and representative fiber lasers are given in Table 2. These manifest 2D Xenes applicability as a versatile 2D broadband SA candidate.

**Table 2:** Performance summary of Q-switched lasers based on 2D Xenes SAs.

2D materials	Fabrication method	Incorporation method	Modulation depth (%)	*λ* (nm)	Duration (µs)	RF (kHz)	Energy (µJ)	Ref.
Antimonene	LPE	SAM	18	2016	2–3.5	–	8.89	[109]
			8	3466.6	3.9–3.7	–	0.22	
Antimonene	LPE	SAM	19.1	2865	1.74–4	59.52–156.2	0.72	[110]
Antimonene	LPE	Film	13.06	1559.63	1.58–9.23	25.3–76.7	37.9 nJ	[111]
Antimonene	Vapor deposition	D-shaped fiber	7.1	1561.9	983 ns	23.26	–	[112]
			7.1	2789.5	824 ns	201.5	–	
Antimonene	LPE	Tapered fiber	–	1557	1.31	20–50	–	[113]
Antimonene	LPE	Quartz substrate	3.5	946	208.8 ns	268.3	0.31	[114]
			–	1064	129 ns	569.1	0.23	
			–	1342	48.33 ns	28.65	1.36	
Antimonene	LPE	Film	11.63	1558	1.42–5.1	25.6–124.1	54 nJ	[115]
Antimonene	LPE and synchronous fluorination	–	–	1064	0.33–0.89	388.8–733.1	–	[116]
Antimonene	PVD	Film	22	1947	4.9–11.6	14.5–23.5	0.12	[117]
Bismuthene (QDs)	LPE	Quartz substrate	18.1	1060	0.15	424	0.26	[118]
			5.1	1030	0.15	457	0.26	
Bismuthene(QDs)	LPE	Quartz substrate	–	2 µm	0.44	94	4.5	[116]
Bismuthene(QDs)	LPE	Sapphire substrate	8.7	1341	0.51	63–135	0.91	[119]
Silicene	LPE	Film	20.1	1567.1	2.32–5.47	11.1–58.7	–	[120]
Silicene	LPE	Quartz substrate	18.9	946.6	0.2–0.4	166.6–294.5	0.55	[103]
			17	1064.3	0.1–0.25	319.8–587	0.22	
			–	1341.8	0.11–0.30	238.5–570	0.14	
Tellurene	LPE	Film	3.2	605	0.17	208	0.24	[105]
			–	639	0.16	331	0.19	
			–	721	0.12	214	0.25	
Tellurene	Green chemical method	–	4	1595	2.85	44	50 nJ	[121]
Tellurene	LPE	Sapphire substrate	6.3	1064	0.1	535.8	0.26	[122]
			–	1.3 µm	0.18	257.1	0.44	
Tellurene	LPE	Film	12	1341.6	0.95	117	2.25	[123]
Tellurene	LPE	Film	0.97	1563.7	5.196–8.915	15.92–47.61	–	[124]
Tellurene-BP	LPE	–	13.6	1064.3	0.44	71	4.3	[125]
			12.6	1954.4	0.45	65	15.95	
			10.3	2790	0.26	90	13.89	
BP	–	D-shaped fiber	–	1550	9.35	4.43–18	28.3 nJ	[126]
BP	LPE	SAM	15	2.8 µm	1.18	39–63	7.7	[127]
BP	ME	Film	1.94	–	3.4	26–76	–	[128]
BP	LPE	Optical deposition	24	1912	0.73	69.4–113.3	0.63	[129]
BP	LPE	Film	14.3	1561.21–1564.16	2.98–25.10	10.348–30.098	0.28	[130]
BP	LPE	Film	–	635.4	0.38	108.8–409.8	–	[131]
BP	LPE	Film	–	1550	1.73–4.44	16.72–30.71	47.6 nJ	[132]
BP	LPE	Tapered fiber	50.94	1563.1	91 ns–890.7 ns	15.76–295.98	16.5–21.1 nJ	[133]
BP	ME	Film	7	1550	7.04–20.75	9.1–44.33	0.13	[134]
BP	ME	Fiber endface	7	1069.4	12.2	26.1	0.33	[135]

*λ*: central wavelength; RF: repetition frequency; ME: mechanical exfoliation; LPE: liquid-phase exfoliation; PVD: physical vapor deposition; SAM: saturable absorber mirror.

### 5.2 Mode-locked operation

Different from Q-switched lasers, mode-locked lasers can realize the pulse duration in sub-ps level, in recent years, passively mode-locked lasers based on 2D Xenes attracted increasing interest in applications of advanced materials processing, medical diagnosis and treatment, military systems, and optical communication due to their giant superiority stemming from the merits of possessing higher peak power and shorter pulse duration.

**Figure 11:**
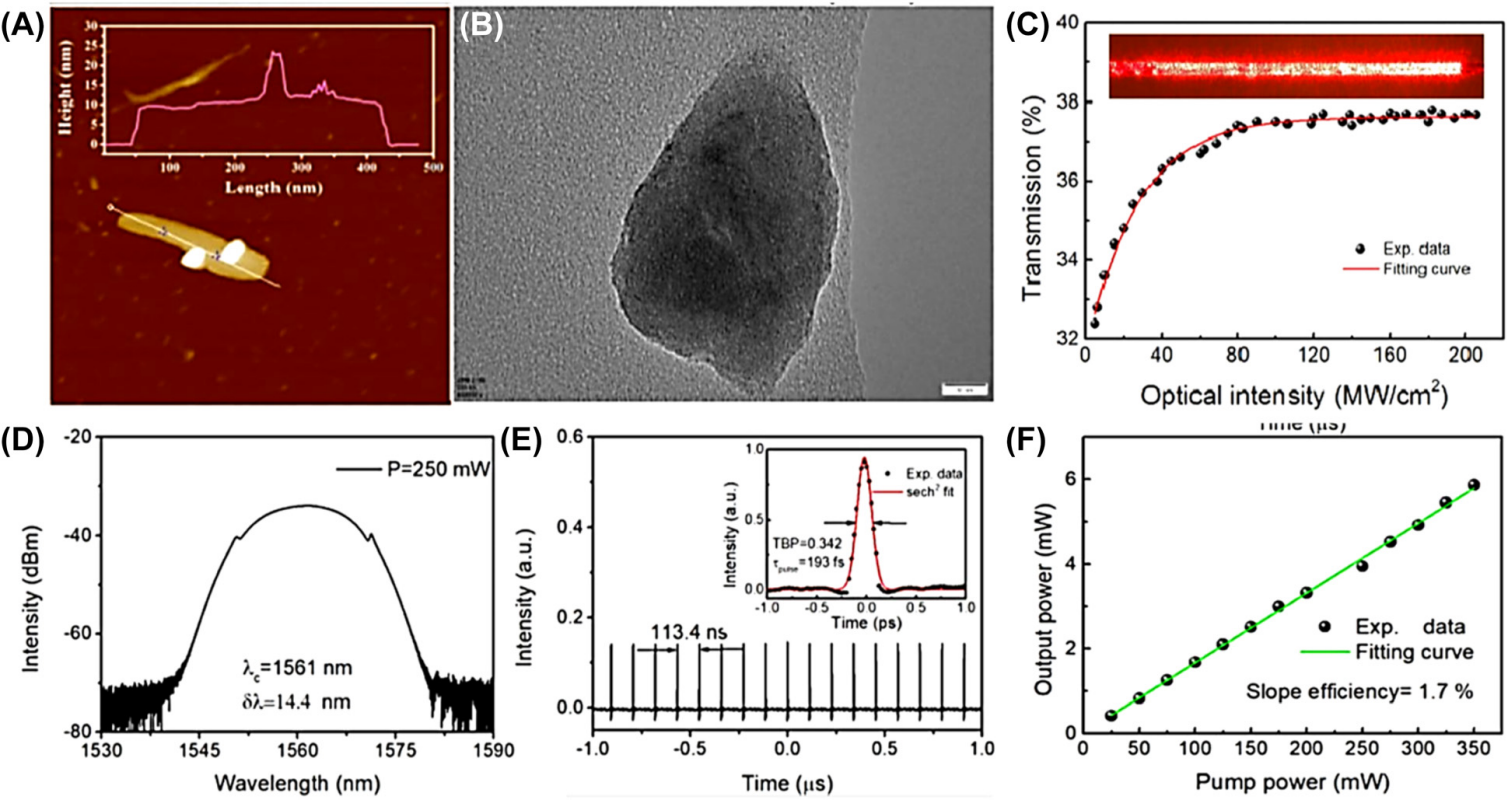
Material characterization: (A) AFM image. (B) TEM image. (C) The nonlinear saturable absorption curve of the microfiber-based bismuthene device (Inset: its corresponding red light image). Mode-locked characteristics of an EDFL based on bismuthene SA: (D) output optical spectrum. (E) Its corresponding oscilloscope trace (inset: the autocorrelation trace). (F) The output power versus the pump power of the soliton pulse. Reproduced with permission [166].

Passively mode-locked operation based on 2D Xenes materials have been adopted to realize pulsed operation due to their more compact in geometry and simpler in setup [163], [164], [165], [166], [167], [168], [169], [170], [171], [172], [173], [174], [175], [176], [177], [178], [179], [180], [181], [182], [183], [184], [185], [186], [187], [188], [189], [190], [191], [192], [193], [194], [195], [196], [197], [198], [199], [200], [201], [202], [203], [204], [205], [206], [207], [208], [209], [210], [211], [212]. Passively mode-locked lasers as an effective tool to investigate soliton dynamics and the related nonlinear phenomenon have received intense attention in recent years. SAs were mainly used to achieve traditional soliton operations with ps or fs level pulse width and relatively low pulse energy limited by the soliton area theorem [98]. The pulse energy of the traditional soliton fiber laser is generally limited by optical wave breaking (the energy of pulses is limited to 0.1 nJ) [215, 216]. Generally, attaching the SA onto the facet of fiber connector is the main way to get ultrafast optical modulator, but it is easy to be damaged by the high energy density of ultrafast pulse. Fortunately, utilize evanescent field interaction between fiber and material provided an approach for solving this issue [218]. For instance, Guo et al. demonstrated a sub-200 fs soliton mode-locked Er-doped fiber laser (EDFL) using a microfiber-based bismuthene SA for the first time, as is illustrated in Figure 11, stable soliton pulses centered at 1561 nm with the shortest pulse duration of about 193 fs were obtained [166]. In 2017, Song et al. discovered the nonlinear optical properties of few-layer antimonene for the first time, through liquid-phase exfoliation and fabricate it as antimonene decorated microfiber with tapered fiber by using evanescent field optical deposition, mode-locked pulses centered at 1.55 µm had been obtained with a pulse width of ∼550 fs, obvious Kelly sideband was observed.

**Figure 12:**
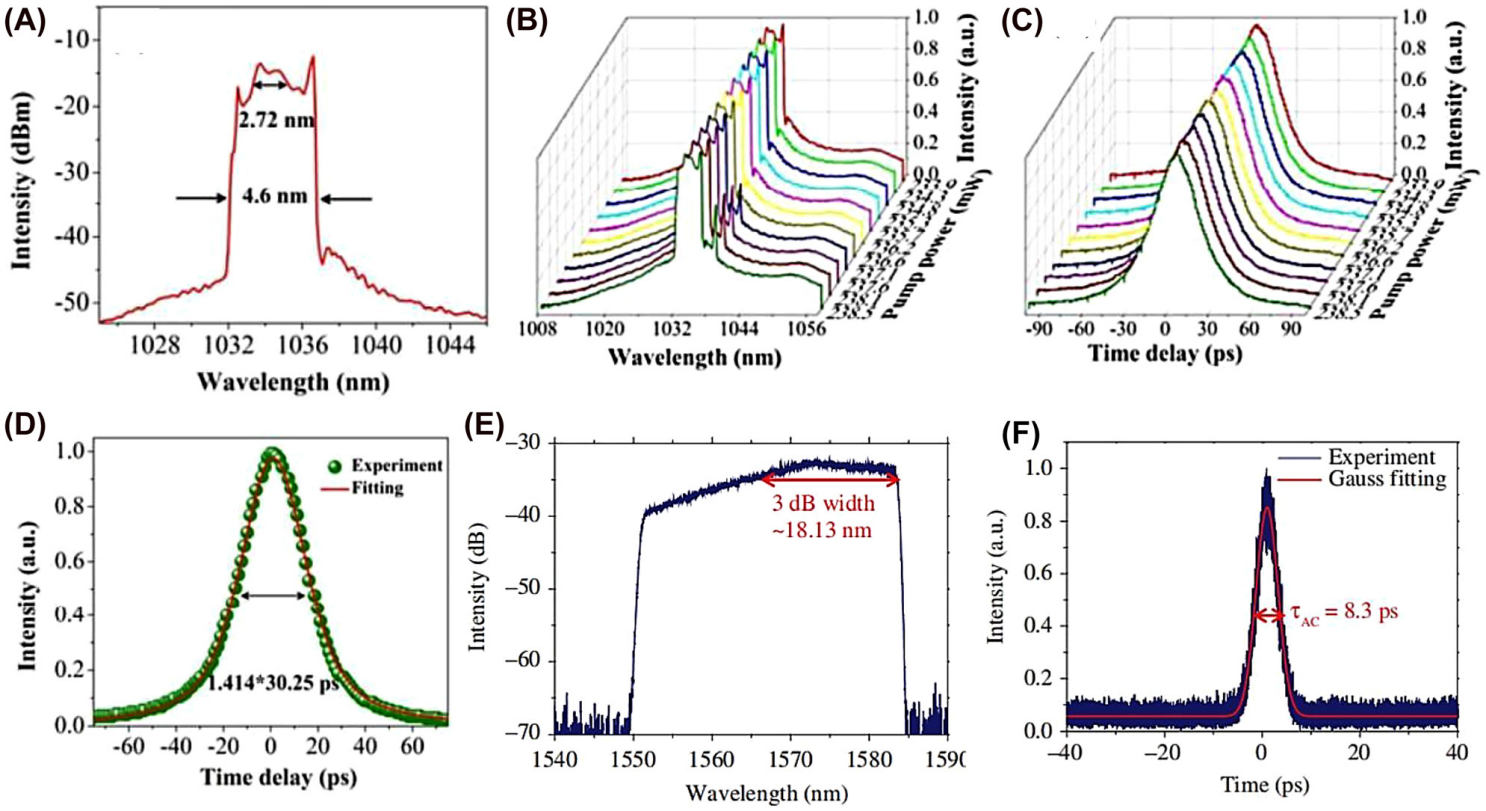
DS characteristics. (A) Typical spectrum of the DSs with center wavelength of 1034.4 nm. (B) The spectral evolution versus the pump power. (C) The time delay evolution versus the pump power. (D) Autocorrelation trace. Reproduced with permission [164]. Output characteristics of tellurene-based SA mode-locked operation. (E) Emission optical spectrum. (F) Autocorrelation traces. Reproduced with permission [98].

High energy optical pulse has attracted extensive attention because of its wide application in optical sensing, optical frequency measurement and data query. Among them, dissipative soliton (DSs) in passive mode-locked lasers have been widely developed in recent years, because they increase the pulse energy of lasers by several orders of magnitude compared with the traditional soliton pulse. It is found that in the normal dispersion state, the laser generates DSs is a balance of the self-phase modulation (SPM) cavity dispersion, Kerr nonlinearity, spectral filtering effect, and cavity loss [217]. Its dynamics is controlled by cubic-quintic GLE. So far, DSs has demonstrated the use of various schemes in lasers. A part of typical characteristics of dissipative soliton are shown in Figure 12. In 2020, based on tellurene as SA, Zhang et al. achieved large-energy mode-locked operations in an all fiber EDFL, both DSs and noise-like pulses were obtained, for dissipative soliton operation, the maximum average output power, pulse width, and largest pulse energy are 23.61 mW, 5.87 ps, and 1.94 nJ, respectively [98], the obtained results provide an enhanced understanding of dynamics of the dissipative soliton and noise-like pulses. Interestingly, thanks to the narrow bandgap of bismuthene and tapered fiber structure, a special kind of noise-like multipulses have been obtained in Yb-doped fiber laser by Feng et al. [165], the saturation intensity and modulation depth of bismuthene SA are about 2.4 MW/cm^2^ and 1%, respectively.

High-damage threshold SA provides the assistance for harmonic mode-locked laser since multiple pulses rearrange themselves in a regular position and the pulse repetition rate is the multiple of the fundamental repetition rate at the high pump regime. In 2017, Pawliszewska et al. reported a mode-locked in Ho-doped all-fiber laser based on BP SA at the central wavelength of 2094 nm [202], 10th harmonic (290 MHz) was obtained, and this is the first demonstration of mode-locked operation in a Ho-doped fiber laser with the a BP SA. In mid-infrared band, Liu et al. succeed in developing a chemically synthesized tellurium nanocrystals (Chem-Te) thin film by liquid phase peeling technique, and demonstrate this material is an effective SA for realization of stable mode-locking in an all-fiber structured Tm-doped laser cavity in 2020, the second-order and third-order harmonic mode-locked pulses train of this fiber laser were also observed, this is the first report on the novel Chem-Te material saturable absorption property realization in 2 μm-band fiber laser system [178]. Xu et al. demonstrated a dual-wavelength harmonic mode-locked EDFL based on a bismuthene SA with large magnitude of third-order nonlinear susceptibility and high carrier motility recently [213], 1st to 20th harmonic mode-locked pulses were obtained when the pump power increased from 43.2 to 201.5 mW, and a 52nd harmonic dual-wavelength pulse (corresponding to the repetition of 208 MHz) was also been obtained when the pump power was larger than 408 mW. The harmonic mode-locking lasers based on 2D Xenes materials have been developed as an important technique to increase the pulse repetition rate [104, 178, 202, 213].

**Figure 13:**
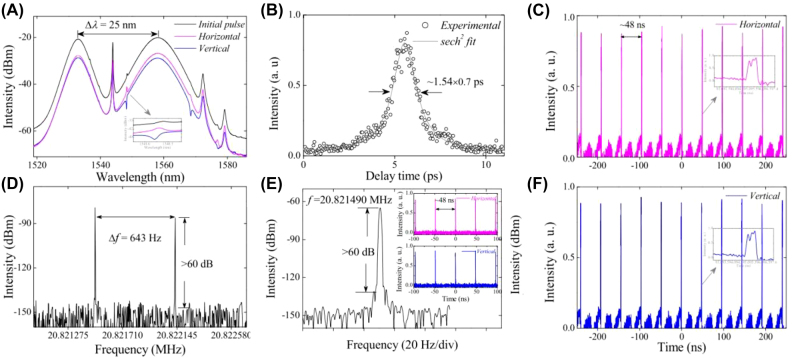
Mode-locked characteristics of an EDFL based on BP SA: (A) Optical spectrum. (B) Autocorrelation traces. (C and F) Polarization-resolved oscilloscope traces of dual-wavelength conventional solitons. (D) RF spectrum. (E) RF spectrum with a span of 100 Hz. Reproduced with permission [190].

Multiwavelength ultrafast fiber lasers have attracted considerable attention for many applications such as in laser radar systems, optical sensors, biomedical research, and high-bit-rate wavelength-division-multiplexing (WDM) optical communication. In recent years, multiwavelength mode-locked operation are also realized based on 2D Xenes [108, 126, 154, 190, 199, 214]. Figure 13 shows the characteristics of dual-wavelength vector solitons in an EDFL based on BP SA achieved by Yun [190], the dual-wavelength centered at ∼1533 and ∼1558 nm with the bandwidths of ∼3.7 and ∼6.9 nm simultaneously, this work reveals the possibility of few-layered BP as broadband SAs in vector soliton pulses generation. In 2019, Wang et al. reported a switchable and tunable multi-wavelength emissions in pulsed Yb-doped fiber lasers with BP SA and polarization-maintaining fiber Bragg gratings at 1063.8 and1064.1 nm [214]. In addition, a three synchronous wavelengths mode-locked EDFL at 1557.2, 1557.7, and 1558.2 nm based on BP SA was realized by Zhao et al. [108], this is the first report about the application of BP as an SA for building a multiwavelength synchronous mode-locked fiber laser.

**Figure 14:**
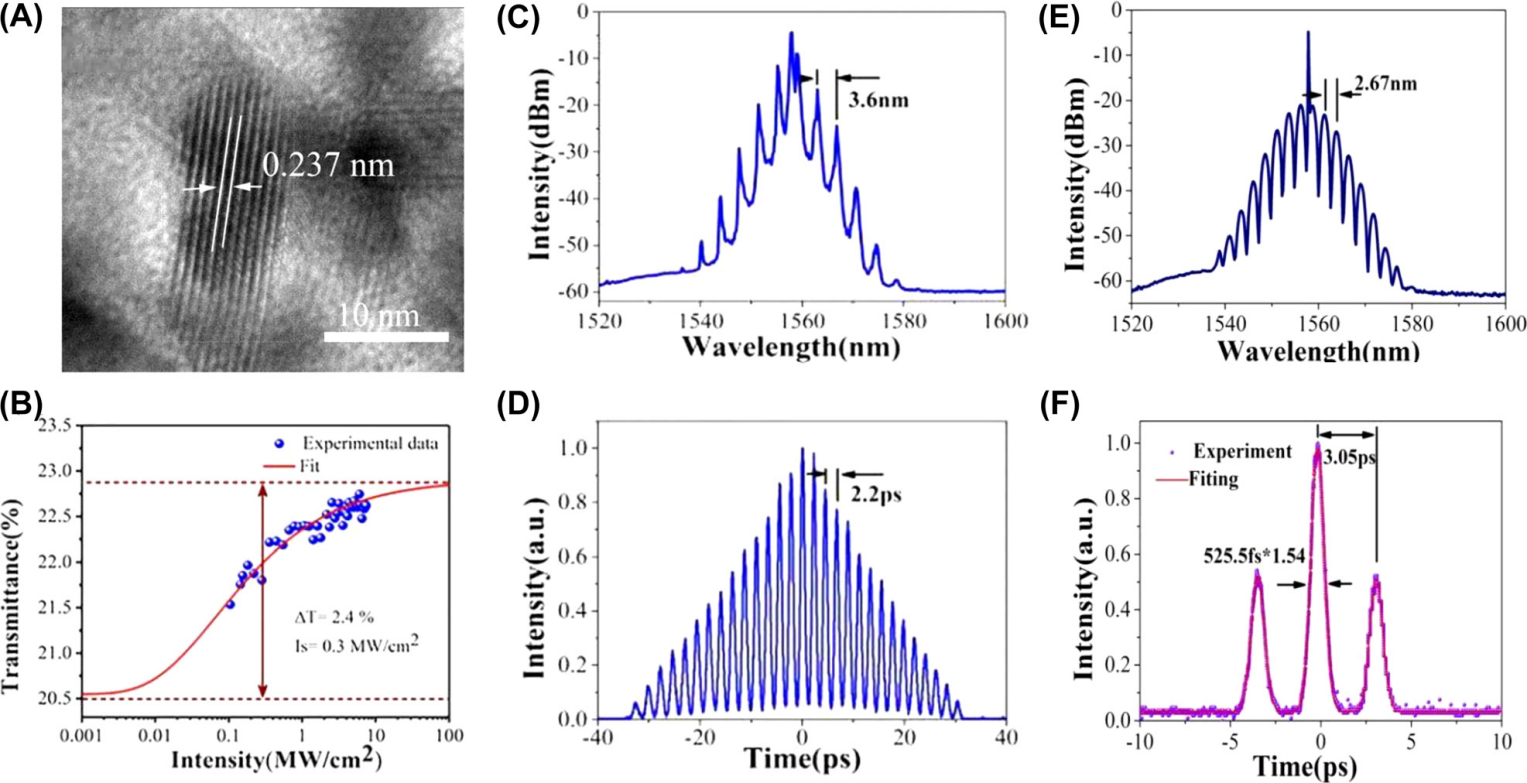
Material characterization: (A) HR-TEM image. (B) Nonlinear transmittance. Soliton molecules mode-locked characteristics. (C) The spectrum with fourteen soliton molecule. (D) Autocorrelation trace. (E) Optical spectrum. (F) Autocorrelation trace of the bound-state. Reproduced with permission [104].

In the laser mode-locked process of 2D Xenes materials, bound soliton pulses are also observed [104, 173, 180]. For example, Liu et al. obtained the soliton molecule with a repetition rate of 10.36 MHz in an EDFL based on BP QDs SA, which was fabricated by using tapered fiber, both traditional soliton and noise-like pulse were also investigated in this work [180]. In 2019, Wang et al. reported on fs soliton molecules generation in EDFL based bismuthene SA, as presented in Figure 14, dual-pulses, octonary-pulses and fourteen-pulses soliton molecules with tightly and loosely temporal separation can be achieved due to the outstanding nonlinear effect and semimetal of the bismuthene, the separations of bound pulses have small variations [104]. It was founded that different spectral modulations were caused by the different widths and peak intensities of the constructed solitons within the bound state.

**Figure 15:**
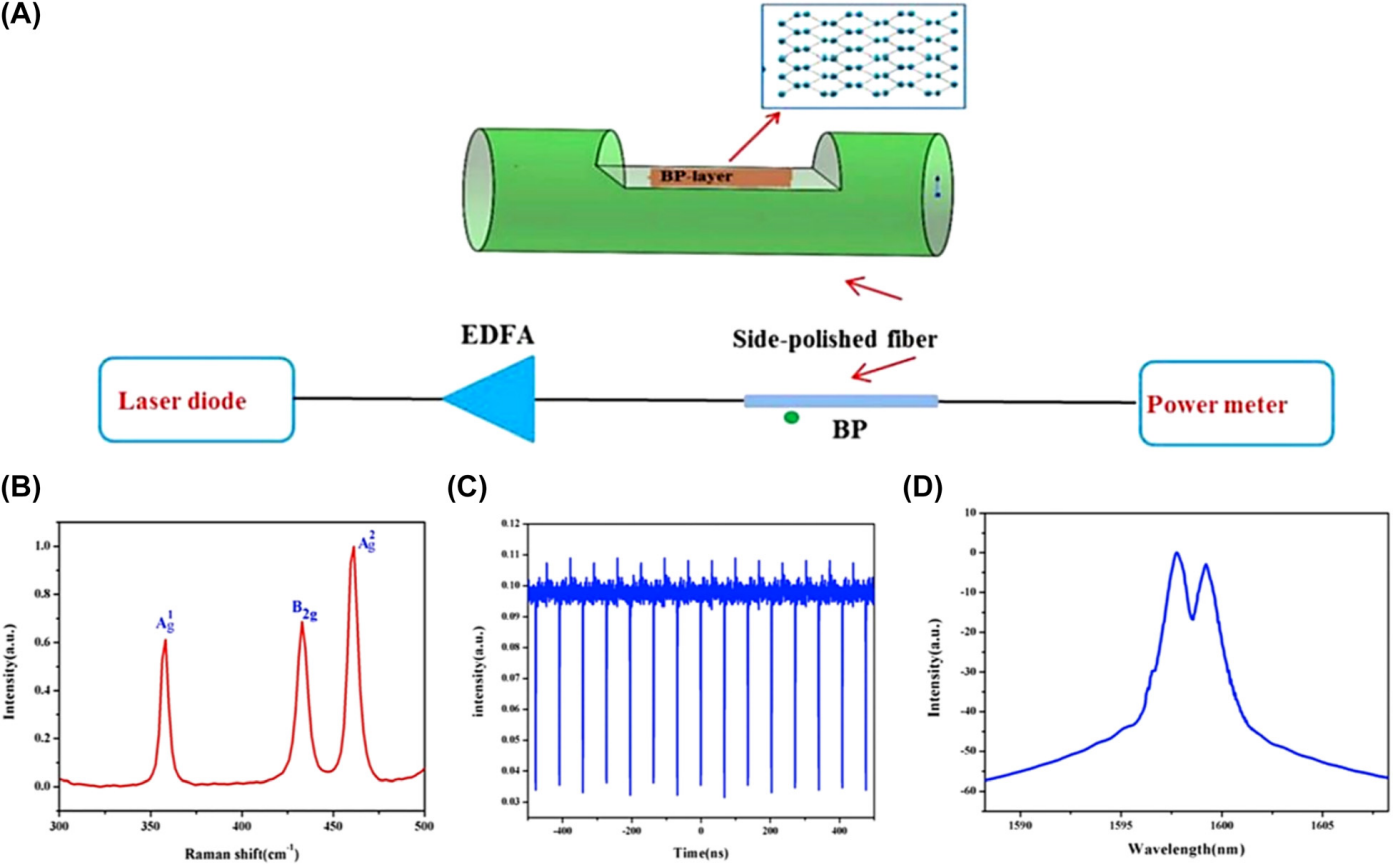
Material characterization: (A) Experimental setup for optically-driven deposition of BP on side-polished fiber (EDFA:erbium-doped fiber amplifier) and the schematic structure of the BP-based nonlinear device and the crystal structure of BP-layer with top view. (B) The Raman spectrum of BP. (C) Dark solitons mode-locked operation. (D) Optical spectrum. Reproduced with permission [210].

Notably, dark solitons were also observed in the mode-locked EDFL based on 2D Xenes materials. The mechanism of generating dark solitons in a fiber laser with a net anomalous dispersion can be explained by domain-wall theory, in which two leasing beams originated from two Eigen operation states of fiber laser are coupled incoherently with each other. For example, Liu et al. demonstrated the dark solitons with a repetition rate of 14.68 MHz in an EDFL based on a BP SA, as depicted in Figure 15, BP SA were deposited on side-polished fiber by optically-driven deposition method [210], this obtained results provided an important understanding of dynamics of the dark soliton.

It is found that compared with Kerr lens mode-locked technology, 2D materials based SAs has more advantages such as self-starting and alignment-free. For ease of application, 2D materials are usually coated on high reflectivity mirrors and then used as cavity mirrors. Notably, a mode-locked Nd:GdVO_4_ bulk laser operating at 1.34 μm was realized by using BP SAM by Sun et al. in 2017 [197], the pulse duration was 9.24 ps which is the shortest among the mode-locked 1.34 μm neodymium lasers ever obtained with other 2D materials SA.

Table 3 only counts the sub-ps level mode-locked lasers based BP as SA, there are many other meaningful works were listed in Ref [200], [201], [202], [203], [204], [205], [206], [207], [208], [209], [210], [211], [212]. These findings deepen the understanding of nonlinear dynamics of soliton operation, and further demonstrate that the 2D Xenes based SA may operate as a high-performance photonic device for exploring nonlinear optical phenomena.

**Table 3:** Performance summary of mode-locked fiber lasers based on 2D Xenes SAs.

2D materials	Fabrication method	Incorporation method	Modulation depth (%)	*λ* (nm)	RF (MHz)	SNR (dB)	Duration (fs)	Ref.
Antimonene	Vapor deposition	D-shaped fiber	7.1	1562.64	–	64.7	753	[112]
Antimonene	LPE	Fiber endface	9	1564	2.16	–	1.73 ns	[102]
Antimonene	LPE	Tapered fiber	3.96	1557.68	10.27	50	552	[113]
Antimonene	PVD	Film	–	1914	8.65	77	64 ps	[117]
Antimonene	PVD	Film	23	1559	0.99	70	3530	[163]
Bismuthene	SCE	Tapered fiber	2.2	1034.4	21.74	45	30.25 ps	[164]
Bismuthene	–	Tapered fiber	1	1035.8	21.74	29.6	54.19 ps	[165]
Bismuthene	SCE	Tapered fiber	5.6	1561	18.85	–	193	[166]
Bismuthene	SCE	Tapered fiber	2.5	1531	4	56.54	1300	[167]
Bismuthene	SCE	Tapered fiber	2.5	1555.7	4	30	1300	[168]
Bismuthene	SCE	Tapered fiber	2.03	1559.18	8.83	55	652	[169]
Bismuthene	SCE	Tapered fiber	–	1557.5	22.74	25	621.5	[104]
Bismuthene	LPE	SAM	–	2030	16.7	–	1 ns	[171]
Se-BP	LPE	–	18.7	1579.4	12	63	686	[172]
Selenene	LPE	Tapered fiber	2.13	1555.67	13.68	65	3100	[173]
Silicene	LPE	Tapered fiber	20	1531.48	3.35	43	937	[174]
Tellurene	Anaerobic bacteria	Film	–	–	18.8	–	1810	[175]
Tellurene	Green chemical	–	4	1566.7	1.87	40	3560	[176]
Tellurene	LPE	Film	27	1556.57	15.45	53	829	[106]
			10.5	1060.16	4.45	64	465.6 ps	
Tellurene	LPE	Tapered fiber	35.64	1558.8	3.327	37.95	1030	[177]
Tellurene	LPE	Film	13.71	1971	11.17	54	890	[178]
Tellurene	LPE	Film	–	1565.58	5.0378	42.3	21.45 ps	[124]
Tellurene	LPE	Film	5.06	1573.97	12.17	55	5870	[98]
			–	1063.97	12.17	55	105.82	
Se-Te	LPE	Tapered fiber	3.436	1555	18.5	60	889	[179]
			–	10,664	18.4	57	11.7 ps	
BP	LPE	Tapered fiber	9	1562.8	10.36	550	291	[180]
BP	ME	Fiber endface	–	1560.5	28.2	65	272	[181]
BP	LPE	Tapered fiber	9	1532–1570	4.96	50	940	[182]
BP	–	–	–	1564.6	3.47	–	690	[183]
BP	LPE	Tapered fiber	8.1	1561.7	5.47	67	882	[184]
BP	ME	Fiber endface	–	1558.7	14.7	56	786	[185]
BP	LPE	–	–	1562	12.5	60	635	[186]
BP	LPE	Tapered fiber	10.1	1549–1575	60.5	68	280	[187]
BP	ME	Fiber endface	4.1	1910	36.8	70	739	[188]
BP	LPE	–	7.75	1560	15.2	65	580	[189]
BP	LPE	Fiber endface	0.35	1558	28.8	60	700	[190]
BP	–	Fiber endface	12.57	1567.2	–	60	538	[191]
BP	ME	Fiber endface	8.1	1550	–	–	720	[192]
BP (QDs)	LPE	Film	4.9	1568.5	15.16	64	787	[193]
BP	LPE	Film	20.1	1859	20.95	60	139	[194]

SCE: sonochemical exfoliation.

**Figure 16:**
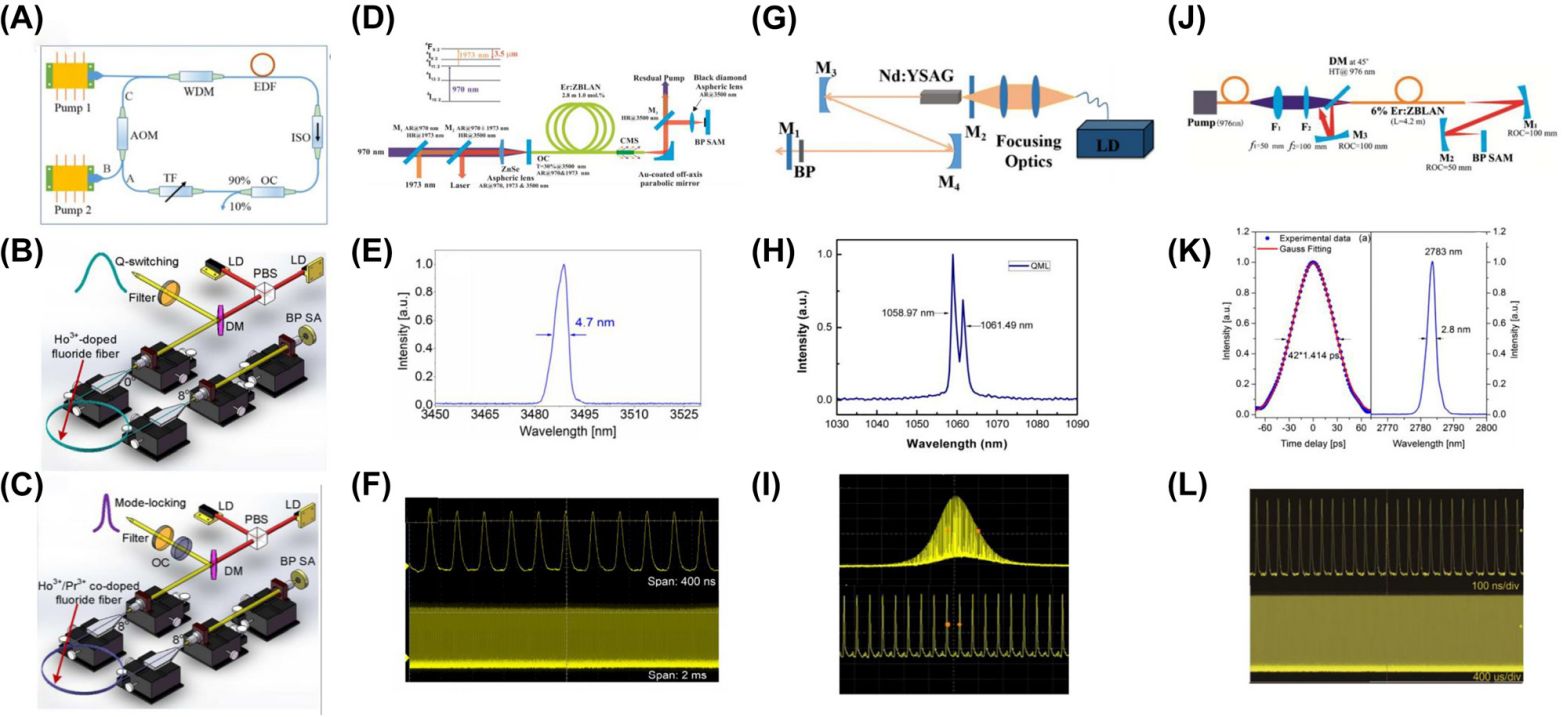
Typical schematic diagram of partial spatial structure lasers: (A) Schematic diagram of actively Q-switched fiber laser by the antimonene AOM setup. Reproduced with permission [219]. (B and C) Experimental setup of pulsed fluoride fiber lasers based using BP SA. Reproduced with permission [149]. (D) Schematic of the BP mode-locked Er:ZBLAN fiber lasers. (E) Mode-locked pulse spectrum. (F) Mode-locked pulse train. Reproduced with permission [150]. (G) Experimental setup for QML Nd:YSAG laser. (H) Optical spectrum. (I) The QML pulse trains. Reproduced with permission [199]. (J) Schematic of the mode-locked Er:ZBLAN fiber laser based BP SA. (K) Autocorrelation trace. (L) Optical spectrum. Reproduced with permission [196].

Furthermore, besides the single scheme, hybrid mode-locked was also demonstrated. Solid state lasers are usually composed of free-space cavity. They are formed by mirrors using doped glass or crystal host materials as solid-state gain media and most commonly applied in science research, military and medical. Figure 16 displays typical schematic diagram of a partial spatial structure lasers. Table 4 summarizes the performance of mode-locked non-all fiber lasers based on BP SAs. Gao et al. reported a passively Q-switched mode-locked (QML) dual-wavelength Nd:YSAG lasers works at 1058.97 and 1061.49 nm based on BP SA, as shown in Figure 16G, the QML operation was realized by designing a Z-type resonator cavity, the duration of mode-locked pulse inside the Q-switched envelop was estimated to be 235 ps with 96.5 MHz repetition rate [199]. In 2016, based on Er:ZBLAN fiber, Qin et al. demonstrated a mode-locked laser with BP SA at the wavelength of 2.8 μm for the first time, Figure 16K presents that the pulse duration was 42 ps [196]. In 3.5 μm spectral region, Qin et al. proposed a mid-infrared Er:ZBLAN fiber laser based BP as SA, the schematic diagram of experiment is shown in Figure 16D, based on BP SAM, both Q-switched and mode-locked operation were realized, these results show that BP has a great potential as mid-infrared SA beyond 3 μm wavelength, which is lacking currently in this wavelength region [150]. In 2020, Yan et al. demonstrated passively Q-switched all-solid-state lasers operating at 1.0, 2.0, and 2.8 μm by using tellurene/BP heterojunctions, their nonlinear optical absorption properties at 1.0, 2.0, and 2.8 μm have been studied by an open-aperture Z-scan method [125]. The experiment results indicate that 2D Xenes especially 2D Xenes based heterojunctions have great potential in the fields of ultrafast applications and other optoelectronic and photonic devices.

**Table 4:** Performance summary of mode-locked non-all fiber lasers based on BP SAs.

2D materials	Fabrication method	Incorporation method	Gain medium	Modulation depth (%)	λ (nm)	RF (MHz)	Duration (ps)	Ref.
BP	LPE	SAM	Nd:YVO_4_	7.5	1064.1	140	6.1	[195]
BP	ME	SAM	Er: ZBLAN	19	2783	24.27	1.414	[196]
BP	LPE	SAM	Nd:GdVO_4_	16	1340.7	58.14	9.24	[197]
BP	ME	Fiber endface	Zr-Er fiber	8	1602	1	3.46	[198]
BP	LPE	Film	Ho/Pr fluoride fiber	41.2	2886.7	13.987	8.6	[199]
BP	LPE	SAM	Er-ZBALAN fiber	7.7	3489	28.91	–	[200]
BP	LPE	Film	Nd:YSAG	11.98	1058.97	96.5	235	[201]

## 6 Conclusions

In this review, we have discussed the ultrafast applications of Xenes beyond graphene. The preparation methods of Xenes and various integration strategies are detailedly introduced at first. Then, according to the materials characterization and nonlinear optical absorption properties we summarize the outcomes achieved by Xenes-based fiber lasers and make classifications based on the characteristics of output pulses. Different from graphene, 2D Xenes materials have outstanding characteristics of tunable bandgap, ultrahigh surface-volume ratio caused by folded structure which have raised widespread concerns in various fields. However, the investigation of 2D Xenes materials SA-based lasers is still in its infancy, as the unique properties of 2D materials can provide fascinating new opportunities and challenges to design the properties through different manufacturing processes because the reliability of these ultrafast optical switches based on 2D nanomaterials are required to be proven to the same extent as existing saturable absorber technology such as SESAM.

Rising enthusiasm in probing varied world of 2D materials constantly promote us to look for novel physical and technological breakthroughs. We believe that 2D Xenes materials could play an important role in future optoelectronic and photonic technologies, particularly as a kind of wideband SA for ultrafast lasers. It is expected that the research in 2D Xenes materials-based all-optical modulation will continue at a fast pace and further exploit practical applications including not only SAs, but also photodetectors, optical modulators, and light-emitting devices.
